# The crosstalking immune cells network creates a collective function beyond the function of each cellular constituent during the progression of hepatocellular carcinoma

**DOI:** 10.1038/s41598-023-39020-w

**Published:** 2023-08-03

**Authors:** Nicholas Koelsch, Faridoddin Mirshahi, Hussein F. Aqbi, Mulugeta Saneshaw, Michael O. Idowu, Amy L. Olex, Arun J. Sanyal, Masoud H. Manjili

**Affiliations:** 1 Department of Microbiology & Immunology, Virginia Commonwealth University School of Medicine, Richmond, VA 23298 USA; 2 Department of Internal Medicine, VCU School of Medicine, Richmond, VA 23298 USA; 3 College of Science, Mustansiriyah University, P.O. Box 14022, Baghdad, Iraq; 4 Department of Pathology, VCU School of Medicine, Richmond, VA 23298 USA; 5 Department of Microbiology & Immunology, VCU Massey Cancer Center, 401 College Street, Box 980035, Richmond, VA 23298 USA; 6 C. Kenneth and Dianne Wright Center for Clinical and Translational Research, Virginia Commonwealth University School of Medicine, Richmond, USA

**Keywords:** Cancer, Immunology

## Abstract

Abundance of data on the role of inflammatory immune responses in the progression or inhibition of hepatocellular carcinoma (HCC) has failed to offer a curative immunotherapy for HCC. This is largely because of focusing on detailed specific cell types and missing the collective function of the hepatic immune system. To discover the collective immune function, we take systems immunology approach by performing high-throughput analysis of snRNAseq data collected from the liver of DIAMOND mice during the progression of nonalcoholic fatty liver disease (NAFLD) to HCC. We report that mutual signaling interactions of the hepatic immune cells in a dominant-subdominant manner, as well as their interaction with structural cells shape the immunological pattern manifesting a collective function beyond the function of the cellular constituents. Such pattern discovery approach recognized direct role of the innate immune cells in the progression of NASH and HCC. These data suggest that discovery of the immune pattern not only detects the immunological mechanism of HCC in spite of dynamic changes in immune cells during the course of disease but also offers immune modulatory interventions for the treatment of NAFLD and HCC.

## Introduction

Hepatocellular cancer (HCC) is an important cause of cancer-related mortality and its incidence continues to rise both in the West and other parts of the world^1^. The rising incidence of HCC has been linked to both the growing problem of obesity and development of nonalcoholic fatty liver disease (NAFLD)^2,3^. NAFLD encompasses both a nonalcoholic fatty liver (NAFL) and nonalcoholic steatohepatitis (NASH). NASH has a greater propensity to progress to cirrhosis^4^. HCC in NAFLD can develop in the absence of cirrhosis but the risk of HCC is greatest in those with NASH and advanced fibrosis^1,4^. The hallmark of NASH is lipotoxic injury to the liver with activation of the hepatic immune system which is linked both to progression to cirrhosis and to development of HCC^5,6^. The mechanisms by which the inflammatory immune responses link to activation of the oncogenic pathways is not fully understood.

Over the last decade, a large body of literature has been generated on the pathways linked to HCC development specifically in the context of NAFLD. Historically, much of the literature has focused on the potential role of one specific pathway or cell type in the genesis of HCC, seeking for a cause-effect direction. The literature on the role of inflammatory cells, cytokines and pathways have also been similarly focused on using highly reductionist approaches. This has however led to somewhat conflicting data from different studies. For instance, while some studies suggest that CD4^+^ T cells could inhibit HCC^7,8^, another study demonstrated that restoration of CD4^+^ T cells alone was not sufficient to prevent HCC^9^. Some reports suggest that Th17 cells inhibit HCC^10,11^, others show that Th17 cells promote liver injury and HCC^12,13^. Also, dual roles for the immune response in inducing liver injury and HCC has been reported^14^. When it comes to cancer therapies, immune tolerance, immune suppression or immune evasion pathways are being targeted without offering a cure for advanced cancers beyond alleviating the symptoms or prolonging patient survival. This is because of focusing on specific cell types or pathways, such as anti-CTLA4 immunotherapy^15^, anti-PD1 immunotherapy combined with targeting FGFR4^16^, CAR T cell therapy^17^, NK cell therapy^18^, tyrosine kinase inhibitors^19^, and a dual CCR2/CCR5 antagonist targeting monocyte and lymphocyte recruitment in NASH^20^, none of which are sole driver of NASH or HCC.

In the Era of information overload, we are beginning to appreciate the limitations of reductionist approaches for understanding the immunobiology of human diseases because of internetwork interactions of immune cells with one another as a system, and with the tissue microenvironment^21,22^. There is growing appreciation that the net biological impact is often more complex and reflects an interplay between multiple processes that are simultaneously ongoing and dynamically changing. Thus, changes in a given inflammatory cell type density and function in the liver may have variable effects based on the context in which it occurs. Emerging data also reveal that immune mechanisms can be better understood through the discovery of mutual cellular interactions creating a dynamic system with a range of outcomes, rather than by seeking for an assumptive cause-effect direction^23–25^. Nevertheless, understanding of the outcome of dynamic changes in immune cell functions through mutual interactions requires a model to discover the collective function. Recently, we demonstrated that analysis of the proportion of immune cell types interacting with one another could discover the mechanism by which the hepatic immune responses function as a system^9,26,27^. Recently, we have reported that the immunological pattern, rather than each immune cell alone, can better explain the immunobiology of NAFLD and HCC^9,26,27^. Recent advances in deep sequencing as well as artificial intelligence and advanced analytic methodologies algorithms for the analysis of big data^22,28,29^ allow assessment of the immune cells interactions and an overall analysis of the collective immune-inflammatory functions of the immunological patterns.

In this study, we sought to discover the hepatic immunological patterns and their collective function orchestrating the progression of NAFLD and NASH to HCC in a diet-induced animal model of NAFLD (DIAMOND)^30^. This animal model has been validated to reflect many features of progressive NASH in humans and was recently included as one of the best mouse models of NAFLD. Upon initiation of a high fat diet with ad lib administration of sugar water, these mice develop fatty liver and steatohepatitis, leading to HCC^26^. The HCC in these mice have been characterized molecularly and we found them to be similar to S1 and S2^30,31^ forms of human HCC, as well as similar clinical manifestations reported in patients with NASH^32^.

## Results

### Progression of NAFLD and HCC are associated with remodeling of the hepatic structural cells, fibroblasts and endothelial cells, associated with shifts from predominant T and B cells to macrophages and monocytes

**Figure 1:**
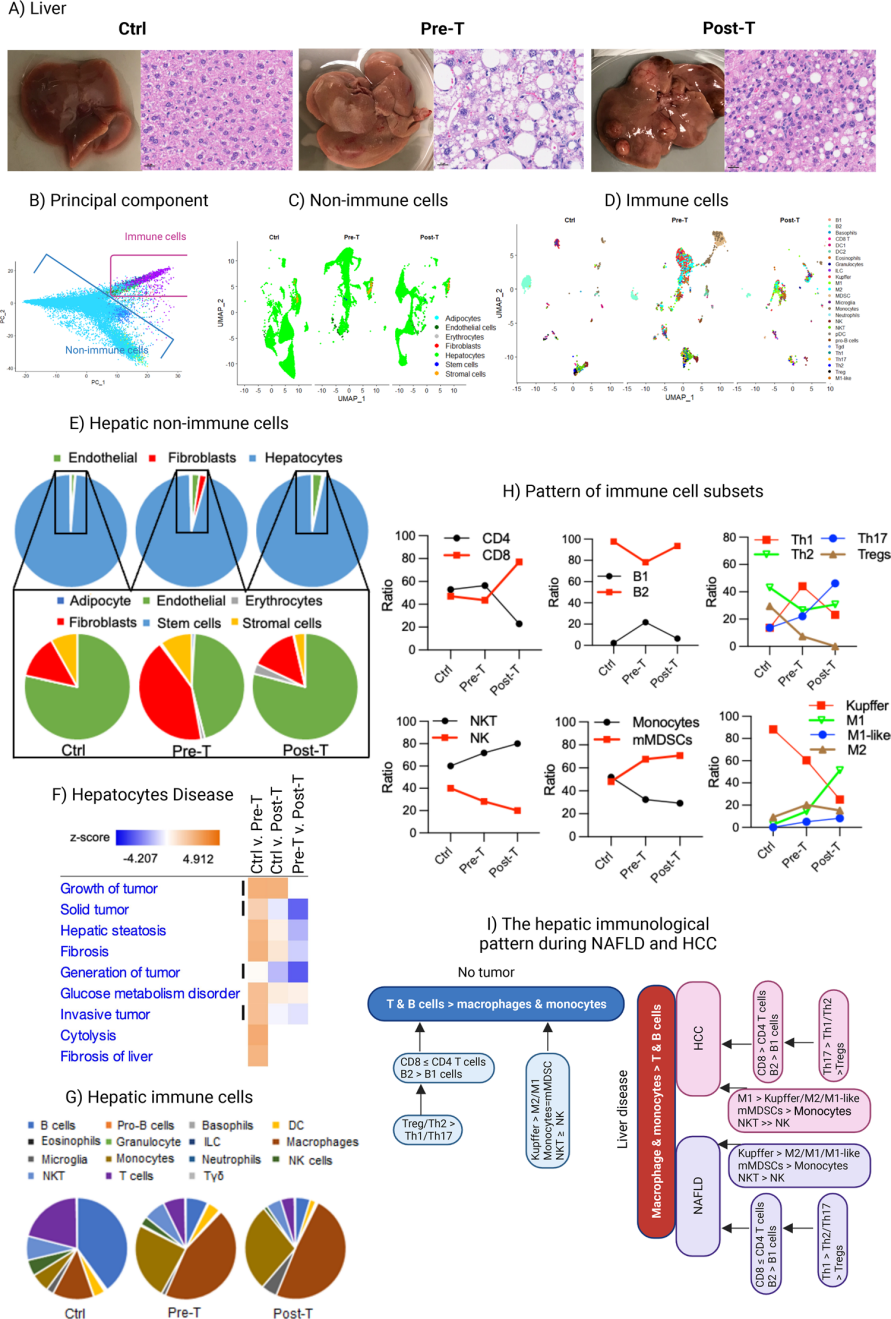
Visualization and pattern assessment of hepatic immune and non-immune cells: (**A**) Livers as well as hematoxylin–Eosin staining of liver specimens collected from the control group (Ctrl) as well as from animals being on a WD prior to tumor development (Pre-T) or after the development of HCC (Post-T). IHC pictures were generated using VectraPolaris 1.0 acquisition software, image size of 16.76 mm × 30.18 mm with the resolution of 25 µm/pixel (40×). (**B**) SingleR annotated cell types after all cells were classified by the ImmGen and MouseRNAseq reference databases. After annotations were complete and compared, the top 20 marker genes of each cell type (immune and non-immune) were passed to PCA to optimize visualization, indicated by separate clustering of immune and non-immune cells due to the use of these genes. (**C**–**D**) UMAP of pooled samples split to show group specific non-immune cells (**C**) and immune cells (**D**). All SingleR annotated cell types and scSorter identified subsets were included in data visualization. (**E**) Pie graphs displaying non-immune cell components in each sample (hepatocytes accounted for over 95% of non-immune cells in each sample, while fibroblasts and endothelial cells primarily comprised the rest. (**F**) IPA analysis of the hepatocyte population portraying disease-related functions and activation z-scores (blue and orange bar) based on DESeq2 results after filtering on a *p*-value < 0.01 and z-scores > 2. Carcinogenesis events are shown using vertical lines. (**G**) Pie graphs showing the composition of immune cells annotated in each sample by ImmGen and MouseRNAseq databases accessed through SingleR. Panels E & G are based on the percentage of all cells in each compartment (innate and adaptive immune cells and non-immune cells) normalized in each for a total of 100%. (**H**) Ratio of immune cell subsets within each population was identified using scSorter (H only includes cells classified as specific subsets of interest from scSorter to normalize each subset of cells to 100%, while removing any “Unknown” cells that were not classifiable to ensure accuracy). (**I**) Multilayered immunological patterns during health and diseases, manifesting super-patterns and inferior patterns were quantitatively analyzed by focusing on the ratios/proportion of immune cells interacting with each other in a network.

In order to determine the hepatic immune regulation during progressive NAFLD and HCC, livers of DIAMOND mice, being on a regular Chow Diet (CD: Ctrl), as well as those from animals, being on a Western Diet (WD) during progressive NAFLD, either prior to the development of tumor (Pre-T) or after tumor development (Post-T) (Fig. 1A), were subjected to single nuclei RNA sequencing (snRNAseq). First, we employed quality control metrics with Seurat to ensure only high-quality cells were used, followed by a dual reference database annotation of cell types through SingleR, in which matched probability scoring implicated accurate cell type annotation (see Section “Materials and methods”; Fig. S1A–C). Marker genes from annotated cell types were passed to principal component analysis (PCA) to optimize visualization of immune and non-immune cells (Fig. 1B), after which we visualized both compartments separately with UMAP (Fig. 1C,D). In order to detect the crosstalking networks or patterns of the hepatic cells and immune cells interacting with each other, the proportions of the hepatic and immune cell types were analyzed. Such analyses revealed a sustained predominance of hepatocytes comprising 95% of all the non-immune cells, and an increased frequency of fibroblasts during progressive NAFLD shifting it from subdominant to dominant compared with endothelial cells, and returning back to subdominant status during HCC (Fig. 1E). Enrichment analysis with the Ingenuity Pathway Analysis (IPA) tool detected carcinogenesis processes as well as steatosis only in hepatocytes preceding the formation of HCC (Fig. 1F). Quantifying the number of cells within the immune cell compartment detected a shift from the pattern of predominant T and B cells (adaptive immunity) to predominant macrophages and monocytes (innate immunity) (Fig. 1G). Such a shift in the hepatic immunological pattern from T and B > macrophages and monocytes towards T and B < macrophages and monocytes was associated with alterations in the proportion of immune cell types. This included an increased proportion of CD8^+^ T cells > CD4^+^ T cells compared with their comparable proportion in the Ctrl group^9,26^ and a shift from predominant Th2 to Th1 and then Th17 cells (Fig. 1H), as well as a shift from predominant Kupffer cells to M1 macrophages, predominant mMDSC, and increased ratio of NKT > NK cells (Fig. 1H). Unknown cells within the macrophage population were classified as M1-like cells based on their similarity with M1 cells, notably their high expression of CD11d (Itgad) (Fig. S2), which is expressed by M1 macrophages during chronic inflammation^33–35^. A graphical summary of the hepatic immunological pattern during health and diseases, manifesting super-patterns and inferior patterns were quantitatively analyzed by focusing on the ratios/proportion of immune cells interacting with each other in a network (Fig. 1I).

### The hepatic immunological pattern dominated by macrophages and monocytes creates a collective function that orchestrates the transition from tissue-protective to liver-damaging and tumor-promoting immunity

**Figure 2:**
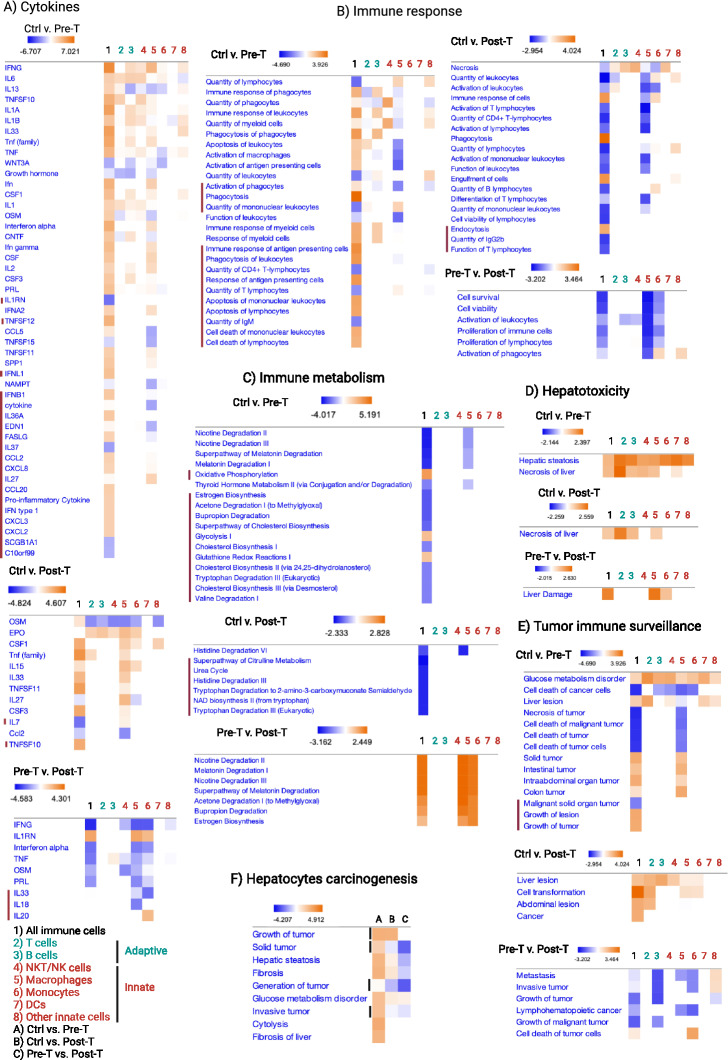
A shift in immunological pattern from a dominant adaptive immunity to innate immunity alters the immune cells function and creates a collective function beyond its cellular constituents: DESeq2 was utilized to assess differences in the entire immune cell population (1) compared to each immune cell type components, including T cells (2), B cells (3), NKT/NK cells (4), Macrophages (5), Monocytes (6), DCs (7), and other immune cells (ILCs, granulocytes, eosinophils, basophils and microglia-like) (8) by using SingleR annotated cell types and passing differentially expressed genes to IPA. Cytokines, immune response pathways and immune metabolism that were only detected by collective immune function analysis (#1) but not the immune cell constituents separately (#2–8) are marked using red line. (**A**) Upstream analysis of comparative groups were performed to detect cytokines. (**B**) Diseases & functions analysis was focused on immune response related functions. (**C**) Metabolic canonical pathways were analyzed to detect immune cell metabolic functions identified across groups. (**D**) Toxic functions were analyzed for the detection of hepatotoxicity related functions when comparing immune cells in each group. (**E**) Diseases & functions analysis was focused on carcinogenesis events to detect tumor immunosurveillance functions in immune cells. (**F**) Hepatocyte populations were subjected to diseases & functions analysis focused on carcinogenesis events; columns represent comparisons, in (**A**) Ctrl vs. Pre-T, (**B**) Ctrl vs. Post-T, and (**C**) Pre-T vs. Post-T. Results from DESeq2 were filtered on a *p*-value < 0.01 and z-score > 2 in IPA for analysis. Carcinogenesis events are shown using vertical lines.

Results from differential gene expression analysis of all immune cells, and individual immune cell types were uploaded to IPA, excluding non-immune cells to increase the sensitivity of detection of predicted immune function, and determine whether the collective immune function is the sum of individual immune cell functions. As shown in Fig. 2, the predicted collective immune function (column 1) was beyond and independent from its cellular constituents (columns 2–8). The predicted collective immune function, but not the immune cell constituents, detected specific inflammatory cytokines being present or absent (Fig. 2A, marked rows including IL1RN, TNFSF12, IFNL1, IFNB1, IL36A, EDN1, FASLG, IL37, CCL2, CXCL8, IL27, CCL20, IFN type I, CXCL3, CXCL2, SCGB1A1, C10orf99, IL7, TNFSF10, IL33, IL18, IL20). Also, it detected increased phagocytosis and antigen presentation, as well as cell death of lymphocytes associated with decreased quantity of T and B cells preceding the formation of HCC (Fig. 2B, marked rows). Genes that affect the quantity of lymphocytes were found to be decreased while genes associated with cell death increased collectively, though they were not detectable for each immune cell type (Fig. 2B, marked rows). Alterations in the predicted collective function of the hepatic immune response were associated with significantly reduced cellular metabolism during the progression of NAFLD and HCC, which again, were predicted as collective immune function but not for each immune cell type (Fig. 2C, marked rows). In particular, oxidative phosphorylation and glycolysis, which were reported to be associated with tumor immune surveillance^36^, were found to increase prior to but not after tumor development only when collective functions of the immune cells were analyzed (Fig. 2C, marked rows). Nevertheless, immune cell metabolism was collectively higher in the Post-T compared with that in the Pre-T group (Fig. 2C, marked rows), which was reflected by the dominance of M1 macrophages, as well as a higher ratio of mMDSCs to monocytes and shifts from predominant CD4^+^ T cells to CD8^+^ T cells (Fig. 1H). Such alterations in the hepatic immune patterns increased their hepatotoxicity (Fig. 2D), as well as their liver-damaging and tumor-promoting functions during the progression of NAFLD (Fig. 2E, marked rows). The predicted functional transformation of the hepatic immune response was further confirmed by the analysis of non-immune cells predicting carcinogenesis events only in hepatocytes (Fig. 2F), as well as the pathways linked to mir-802 being associated with HCC^37–39^ increased only in hepatocytes (Fig. S3A). The predicted significance of mir-802 was detected through the pattern of gene expression changes interacting with mir-802 (Fig. S3B).

**Figure 3:**
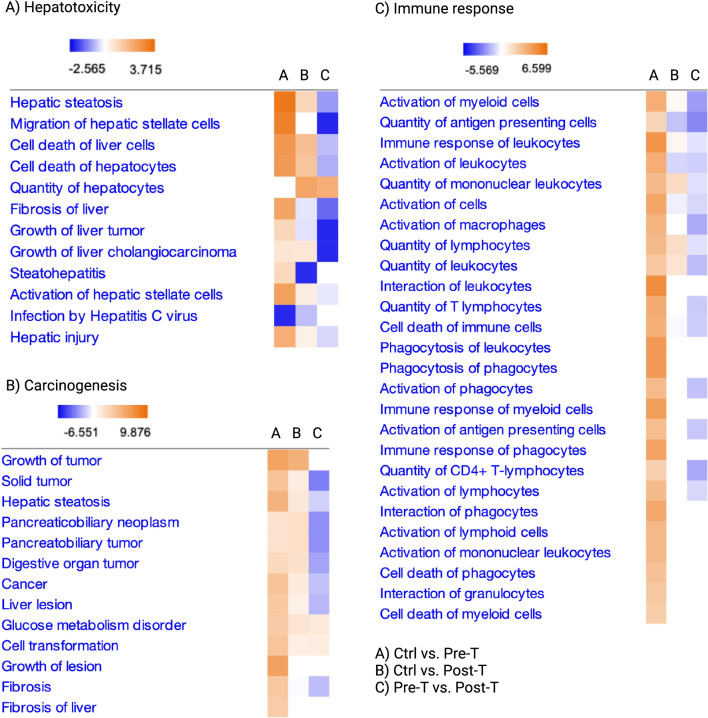
Collective immune function indicates increased inflammatory immune responses associated with liver damage and carcinogenesis events during a WD: All liver cells including the immune [B1, B2, pro-B cells, CD8^+^ T cells, CD4^+^ T cells (Th1, Th17, Th2, Treg), NK, NKT, Tγδ, DC1, DC2, pDC, MDSCs, Monocytes, Macrophages (Kupffer, M1, M2, M1-like), Microglia (microglia-like), Basophils, Eosinophils, Granulocytes, Neutrophils, ILCs] and non-immune cell population [Fibroblasts and Hepatocytes] and subsets from CellChat analysis containing SingleR annotated cell types and scSorter identified subsets were pooled to undergo DESeq2 and analysis in IPA. (**A**) Toxic functions were analyzed for the detection of hepatotoxicity related functions in each group. (**B**) Diseases & functions analysis focused on carcinogenesis events detected across groups. (**C**) Diseases & functions analysis was focused on immune response related functions. Contrasts are as followed and noted in the bottom right legend: Ctrl vs. Pre-T uses Ctrl group as reference, and Pre-T as test; Ctrl vs. Post-T tests the Post-T group against the Ctrl; and Pre-T vs. Post-T compares the Post-T group to the Pre-T as its reference.

In order to determine whether the tumor-promoting collective immune function preceding HCC in hepatocytes, we assessed the pooled SingleR annotated cell types and detected a similar set of events, such as highly upregulated hepatotoxicity and carcinogenesis preceding tumor development (Figs. 3A,B, S3C). These events were associated with inflammatory immune responses and cell death of immune cells altering the hepatic immune system towards tissue-damaging and tumor-promoting functions^40^ (Fig. 3C).

### Structural cells and innate immune cells dominate functional signaling network in the liver

**Figure 4:**
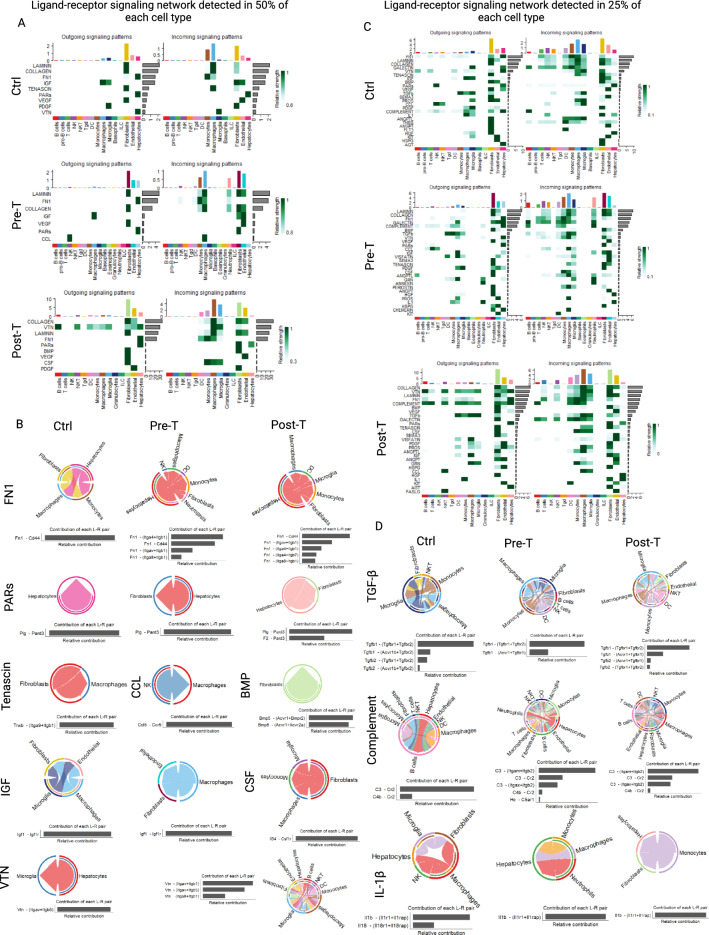
Structural cells and innate immune cells dominate functional signaling network in the liver. (**A**) Heatmaps portraying all signaling pathways found to be significant by the CellChat R package across SingleR annotated cell types, encompassing immune cells, structural cells, and hepatocytes. CellChat analysis parameters were adjusted to use a truncated mean of 50% in order to detect pathways with ligand and receptor genes expressed in at least 50% of cells within annotated cell types, while only mapping significant pathways (*p*-value < 0.05) for each sample to show the cellular sources of signals (outgoing) and those receiving signals (incoming) based on the CellChat database of known ligand-receptor pairs in Ctrl (upper panel), Pre-T (Middle panel), and Post-T (lower panel). (**B**) Chord diagrams of shared signaling pathways ain all groups (FN1 and PARs), unique signaling pathway in each group (Tenascin, CCL, BMP, CSF) as well as shared signaling pathways in the Ctrl and Pre-T (IGF) or Ctrl and Post-T (VTN) groups. (**C**) Heatmaps portraying all identified significant signaling pathways by CellChat using default parameters (25% of cells expressing genes in each cell type. (**D**) Chord diagrams showing three signaling pathways shared by all groups. All figures were made through the use of CellChat version 1.5.0, and heatmaps in (**A**) and (**C**) used the dependent software ComplexHeatmap version 2.15.1 (https://github.com/jokergoo/ComplexHeatmap), while the chord diagrams in (**B**) and (**D**) used the dependent software circlize version 0.4.16 (https://github.com/jokergoo/circlize).

Because of major shifts in the pattern of innate immune cells and structural cells during a WD (Fig. 1), we sought to determine their contribution in the hepatic ligand-receptor signaling network compared with those of adaptive immune cells. First, we focused on the ligand-receptor pathways in which 50% of the cells within each cell type were involved. We found that in all cohorts, fibroblasts and hepatocytes appear to send the majority of signals while fibroblasts, endothelial cells and macrophages dominated the incoming signals (Fig. 4A). In the post-T group, monocytes also dominated the incoming signals (Fig. 4A). Some of these pathways such as FN1 and PARs remained active in all groups, but by targeting different cells in each group (Fig. 4B). Tenascin, CCL, BMP or CSF were uniquely involved in the Ctrl, Pre-T, and Post-T groups, respectively (Fig. 4B). IGF1 remained active in the Ctrl and Pre-T groups while VTN was active in the Ctrl and Post-T groups, yet, targeting different cells in each group (Fig. 4B). A default program analysis focusing on 25% of the cells within each cell type being involved in the hepatic signaling network revealed the appearance of adaptive immune cell (B and T cells) contributing in the signaling network, to a lesser extent than innate immune cells (Fig. 4C). Analysis of the ligand-receptor signaling interactions showed different functional signaling or receptor targeting of the same ligands in each group. For instance, TGF-β showed modulatory effects in the Ctrl group by promoting and inhibiting TGF-β signaling through Tgfbr1/Tgfbr2 and Acvr1b/Tgfbr2, respectively (Fig. 4D). In the Pre-T and Post-T groups, no TGF-β inhibitory signal (Acvr1b/Tgfbr2)^41^ was detected (Fig. 4D). A strong inhibition of complement activation by B cells through CR2 interaction with C3 or C4b was evident in the Ctrl group, and it was switched to complement activation during a WD by the involvement of Itgam/Itgb2 and Itgax/Itgb2 receptor^42^ (Fig. 4D). In the Ctrl group, IL-1β was produced by macrophages and microglia-like targeting fibroblasts, microglia-like, NK cells and hepatocytes, while it shifted towards targeting only hepatocytes in the Pre-T group or hepatocytes and fibroblasts in the Post-T group (Fig. 4D).

### The same immune cell types and cytokines manifest different functional signaling on immune cells and structural cells depending on the dominance of the innate or adaptive immune response

**Figure 5:**
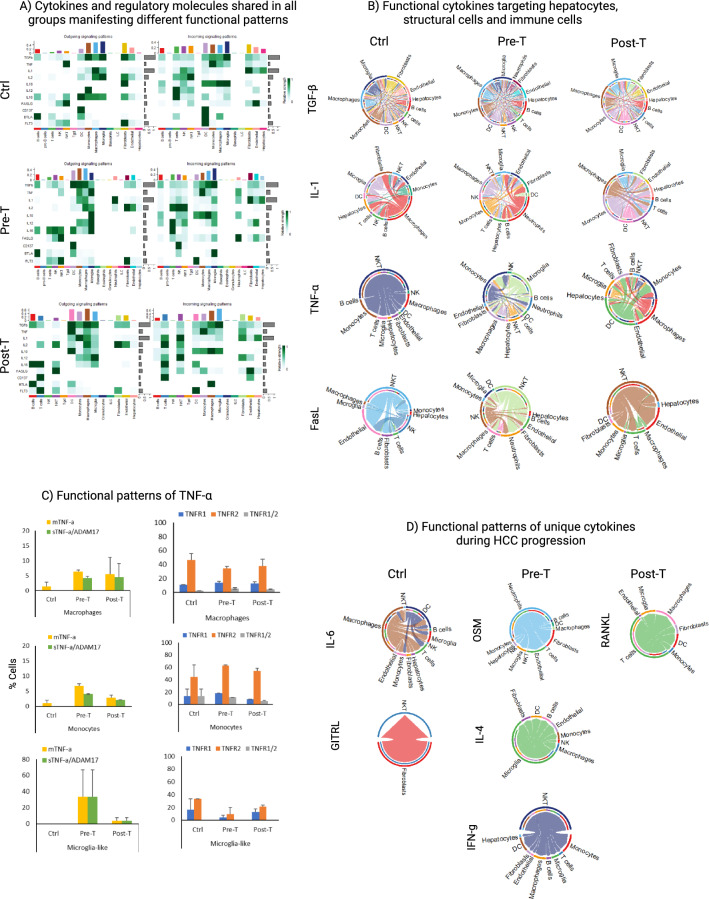
Functional signaling molecules being present in all groups exhibit different patterns of signaling interactions during HCC progression. (**A**) Heatmaps portraying only selected immunologically relevant signaling and regulatory molecules identified by CellChat with analysis parameters adjusted to use a truncated mean of 2.5%, in order to detect functional signaling pathways in reduced numbers of adaptive immune cells during a WD. (**B**) Chord diagrams showing the directionality of functional cytokine signaling targeting hepatocytes (TGF-β, IL-1, TNF-α, and FASL), as well as structural cells, and immune cells. (**C**) Functional patterns of the TNF-α signaling pathway were quantified by assessing the number of cells in each detected cell type involved in the pathway that were TNF-α + (Tnf > 0), TNF-α + /ADAM17 + (double positive and sTNF-α; Tnf > 0 & Adam17 > 0), TNFR1 + (Tnfrsf1a > 0), TNFR2 + (Tnfrsf1b > 0), and TNFR1 + /2 + (double positive; Tnfrsf1a > 0 & Tnfrsf1b > 0); all cells expressing genes are presented as a percentage of the cell type population in each sample. (**D**) Chord diagrams using CellChat for evaluating unique ligand-receptor in the Ctrl (IL-6 and GITRL), Pre-T (OSM, IL-4, and IFN-γ), and Post-T (RANKL). Figures (**A**), (**B**), and (**D**) were made through the use of CellChat version 1.5.0, and heatmaps in A used the dependent software ComplexHeatmap version 2.15.1 (https://github.com/jokergoo/ComplexHeatmap), while the chord diagrams in (**B**) and (**D**) used the dependent software circlize version 0.4.16 (https://github.com/jokergoo/circlize).

In order to determine whether distinct immunological pattern shaped by dominant-subdominant relationship of immune cell types may change functional signaling of the immune cells, all known ligand-receptor interactions were analyzed for each group by including even low frequency cells to cover for the majority of adaptive immune cells as well (Fig. S4). Then, we focused on immune-related pathways, including those shared among three groups or unique to each group. For the shared pathways (Fig. 5A), all functional signaling molecules (TGF-β, TNF-α, IL1, IL2, IL10, IL12, IL16, FASLG) and regulatory molecules (CD137, BTLA, FLT3) were involved in immune cell–cell interaction, though manifesting different signaling pattern in each group. Only four pathways were similarly involved in interactions of immune cells with hepatocyte target cells in all groups (Fig. 5B: TGF-β, TNF-α, IL1, FASLG). Even these four cytokines were dominantly involved in immune cell interactions with one another and with structural cells compared with their interaction with hepatocyte target cells (Fig. 5B). Since TNF-α, which was detected in all groups, can manifest opposing functions depending on its receptors and being soluble or membranous (sTNF-α or mTNF-α), we looked at the expression of ADAM17 for increasing sTNF-α in TNF-α-producing cells^43^ as well as TNFR1/TNFR2 expression^44^ on target cells. We found that macrophages and monocytes were the main target of TNF-α with predominant expression of TNFR2 (Fig. 5C). For the unique cytokines for each group identified in the heatmaps (Fig. S4), IL6 (from macrophages and DCs) and GITRL (from fibroblasts) were detected in the Ctrl group with dominant T and B cells; OSM (from neutrophils), IL4 (from microglia-like), and IFN-γ (from NKT cells) were detected in the Pre-T group; and RANKL (from T cells) was detected in the Post-T group during the dominance of macrophages and monocytes, interacting mainly with other immune cells as well as with structural cells and hepatocytes (Fig. 5D). The dominance of immune cell–cell interactions in all groups was clearly visualized when only immune cells were analyzed (Fig. S5).

**Figure 6:**
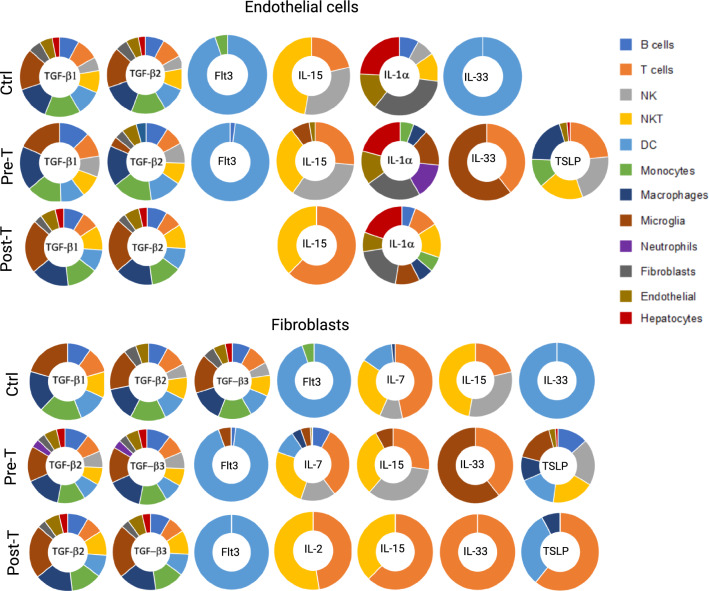
Structural cells are highly influential in the signaling interactions with other immune cells and non-immune cells in the liver. CellChat analyses enabled us to investigate signaling pathways based on the exact ligand and receptor pairs detected in each cell type, using the truncated mean of 2.5% analysis results. We focused on signaling coming from structural cells to immune cells, in which many functional signaling molecules were detected in endothelial cells (upper panel) and fibroblasts (lower panel) across groups.

Given the role of structural cells in regulating organ-specific immune responses^45,46^, we found endothelial cells and fibroblasts affecting mainly the hepatic immune cells rather than hepatocytes (Fig. 6). TGF-β and Flt3 affected mainly innate immune cells while homeostatic cytokines (IL-2, IL-7, IL-15) affected mainly NK or NKT cells as well as T cells. Dominant ratio of fibroblasts over endothelial cells in the Pre-T group not only changed cytokine-mediated communication of fibroblasts with immune cells but also resulted in the production of TSLP by fibroblasts affecting cells of the innate and adaptive immune system in the liver (Fig. 6, bottom rows). IL-33 was engaged with DCs or monocytes in the Ctrl group, as well as with T cells and microglia-like in the Pre-T group, or only with T cells in the Post-T group. Restoration of endothelial cells dominance in the Post-T group, did not restore their functional signaling pattern compared with those in the Ctrl group (Fig. 6). IL-1α was the only inflammatory cytokine from endothelial cells which mainly affected hepatocytes and structural cells in the liver.

### Innate immune cells producing IL-1β are associated with the promotion of HCC

**Figure 7:**
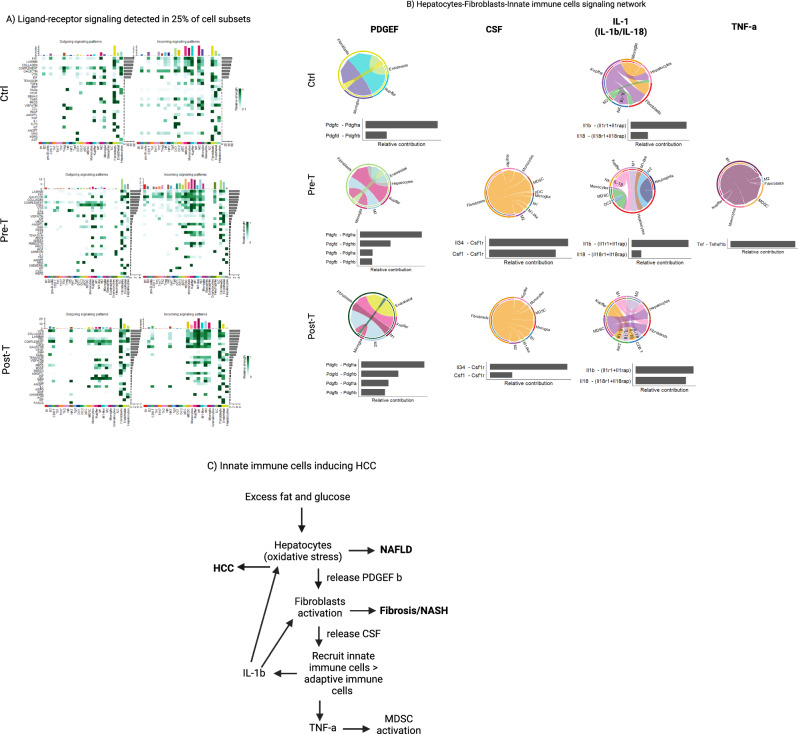
Innate immune cells are central players in promoting HCC. (**A**) Heatmaps portraying all identified significant signaling pathways by CellChat using default parameters (25% of cells expressing genes in each cell type. (**B**) Chord diagrams showing signaling pathways among hepatocytes, structural cells and innate immune cells in all groups. (**C**) A summary of the stepwise signaling communications in the liver during a WD. Figures (**A**) and (**B**) were made through the use of CellChat version 1.5.0, and the heatmaps in A used the dependent software ComplexHeatmap version 2.15.1 (https://github.com/jokergoo/ComplexHeatmap), while the chord diagrams in B used the dependent software circlize version 0.4.16 (https://github.com/jokergoo/circlize).

Given the dominance of the innate immune cells and structural cells in singaling network in the liver, we sought to analyze the signaling pathways while focusing on different innate immune cell subsets interacting with hepatocytes and structural cells (Fig. 7A). We detected the expression of PDGEF c and d isoforms being involved in fibroblasts proliferation and survival in all groups while PDGEF b isoform being involved in fibroblast activation and fibrinogenesis process was detected only during a WD (Fig. 7B). The innate immune cell recruting chemokine, CSF, was produced by fibroblasts only during a WD recruiting macrophages and monocytes into the liver (Fig. 7B). Also, IL-1β was produced by kupffer cells in the Ctrl group wheres it was produced mainly by the innate immune cells targeting hepatocytes in the Pre-T group as well as targeting hepatocytes and fibroblasts in the Post-T group (Fig. 7B). The IL-1 family cytokine, IL-18, targeted NK cells in the Ctrl and Pre-T group while targeting NK cells and CD8^+^ T cells in the post-T group (Fig. 7B). TNF-α was detected only in the Pre-T group produced by M1 macrophages targeting mainly MDSCs and monocytes (Fig. 7B). A summary of the stepwise signaling communications during a WD is showin in Fig. 7C.

## Discussion

The strength of the reductionistic approach lies in a focused and precise analysis of individual components in isolation, yet missing the mutual interconnection and feedback loops between the various cellular components which create a dynamically changing immune system, as well as failure to understand the emergent properties of the immune response as a system. In fact, it misses the forest for the trees. Therefore, there is an urgent need to balance reductionism with a systems immunology approach for a comprehensive understanding of the immune function. A systems immunology approach suggests focusing on cellular interactions and analyzing the feedback loops between the components, understanding the emergent properties or collective function of the immune responses, considering the immune cell interactions with the hepatic structural cells, and finally adopting a holistic perspective that considers the immune responses as a collective function rather than focusing solely on individual components.

By taking a systems immunology approach^28^, we discovered that (i) innate immune cells, M1 macrophages in particular, recruited into the liver are the major player for orchestrating liver fibrosis and progression of HCC (Fig. 7C), (ii) predicted collective immune function in the liver is beyond the function of its cellular constituents, which is absent or undetectable when each single cell type is analyzed separately; (iii) each predicted collective immune function arise from the crosstalking network of immune cells dynamically entangled and interacting with one another and with the hepatic structural cells; it is similar to the behaviors of a flock of birds arising from the interactions between individual birds, rather than from the behavior of any single bird; and (iv) such predicted collective immune function can be understood by the discovery of dominant-subdominant pattern of immune cells interactions as super-pattern as well as inferior-pattern during tumor progression. Specifically, we detected multilayered immunological patterns, extending from super-patterns to inferior patterns, and shifting from the super-pattern of dominant adaptive immunity (B and T cells) > innate immunity (macrophages and monocytes) to the dominant innate > adaptive immunity during the progression of HCC (Fig. 1I). The collective function, which was independent from and beyond the function of its cellular constituents (innate or adaptive immune cells), missed key immune mechanisms associated with the progression of NAFLD and HCC when each immune cell type was analyzed separately. To this end, increased inflammatory cytokines and hepatic immune responses along with reduced metabolism during disease progression were detected only when the collective function of the immune patterns was analyzed, but many of these functional changes were absent for each immune cell type, when they were analyzed separately out of the context of immune cells network. Importantly, oxidative phosphorylation and glycolysis, which were reported as features of tumor immune surveillance^36^, were found to be increased prior to but not after tumor development, only when the collective function of the immune pattern was analyzed. The carcinogenesis events were specifically evident in hepatocytes. These findings suggest that the collective function of the hepatic immune pattern could detect carcinogenesis pathways that precede tumor development, thereby predicting risk of HCC development without having to focus on specific tumor markers. A high incidence of some carcinogenesis events prior to tumor development and their disappearance or downregulation after tumor development is indicative of pathways that are active in normal cells only to alter the normal function of the cells, but they could be absent in already transformed tumor cells. For instance, pathways regulating telomerase reactivation may occur in the early stage of cancer development and then become downregulated or lost as the cancer progresses. Also, some epigenetic modifications may be reversible or dynamic such that they could occur for inducing cell transformation, but they may disappear or change as cancer evolves. Finally, some pre-tumor cells may stimulate angiogenesis to support their own growth, but this may be less pronounced or disappear once a tumor and blood vessels have formed. This is clinically significant because there is no specific marker to predict the risk of HCC in patients with progressive NAFLD and NASH. Therefore, this is the first report showing that detection of the collective immune function can reliably predict risk of HCC progression during NAFLD.

Internetwork analysis of the hepatic cells revealed that structural cells and innate immune cells dominated the ligand-receptor functional signaling network, followed by adaptive immune cells. Importantly, similar ligands interacted with different targets depending on the pattern in which they participated. During the dominance of adaptive immune cells in the Ctrl group, their participation in the ligand-receptor network was less than that of innate immune cells or even less than that of subdominant adaptive immune cells during a WD. This could be because, as we reported previously, only 20% of T cells were of T effector phenotype in the Ctrl group, but the effector phenotypes raised to over 80% during a WD^26^. Importantly, dynamic interactions of the same ligands created different functions, which cannot be explained, when they are analyzed in an isolated fashion by taking a reductionist approach. For instance, while TGF-β was present in the healthy control and those with NAFLD or HCC, its interaction with the activating receptors Tgfbr1/Tgfbr2 and modulatory receptors Acvr1b/Tgfbr2^41^ was present only in the Ctrl group. On the other hand, lack of the modulatory receptor Acvr1b/Tgfbr2 promoted constant activity of TGF-β through Tgfbr1/Tgfbr2 or Acvr1b/Tgfbr1^47^ during NAFLD and HCC. This could explain the TGF-β paradox in tumor inhibition and tumor promotion^48^. Also, complement pathway was detected in all groups but complement activation differed in each group when analyzed in the network of interaction with other immune cells. Although complement activation cannot be drawn from RNA sequencing alone, our findings suggest that dominance of B cells over macrophages in the Ctrl group could result in the CR2-mediated complement inhibitory function of B cells^49^, whereas shifts to dominant innate immune cells during a WD could remove such complement inhibition and promote inflammatory complement activation through the expression of Itgam/Itgb2 and Itgax/Itgb2 receptors by macrophages and monocytes^42^.

By focusing on the signaling pathways among hepatocytes, structural cells, and innate immune cells, we discovered that high fat/sugar diet induces hepatocytes to express the PDGF b isoform, which is typically produced by infiltrating macrophages during inflammation for activation of fibroblasts by signaling through the receptor PDGFRβ, followed by subsequent activation of PI3K/AKT pathways that prompt proliferation, fibrinogenesis, and collagen deposition^50–52^. Importantly, crosstalk between these cell types are implicated in hepatic inflammation, fibrosis, and carcinogenesis^51,53^, while the activation of hepatic stellate cells and fibroblasts in this manner facilitates expression of CSF for the recruitment of innate immune cells in the liver^54–56^. Consequently, the innate immune cells, M1 macrophages in particular, predominated over Kupffer cells and expressed IL-1β to further activate fibroblasts and target hepatocytes, as well as TNF-α to recruit and activate MDSCs. IL-1β is canonically produced by Kupffer cells and infiltrating macrophages during inflammation and can activate these structural cell species^53,57,58^, as well as converting fibroblasts into tumor-associated fibroblasts recruiting innate immune cells^56^. IL-1β was reported to increase carcinogenic events^59^ on hepatocytes during a WD, but it has also been noted to promote steatosis by its effects on hepatocyte fat accumulation^60^. TNF-α signaling through TNFR2 on MDSCs has been identified as a prominent mechanism in the accumulation, persistence, and survival of MDSCs in tumor sites^61,62^, and also for their ability to exert suppressive and tumor promoting functions^63,64^. Importantly, shifts from dominant T and B cells to macrophages and monocytes resulted in the production of TNF-α by macrophages promoting their survival and growth through higher expression of the TNFR2 survival receptor compared with the TNFR1 apoptotic receptor^44,65^ during a WD. Similar observations were made on structural cells with the dominant ratio and signaling of fibroblasts over endothelial cells changing their functional signaling in the Pre-T group. However, restoring endothelial cells dominance pattern did not restore their functional signaling to those in the Ctrl group. This is consistent with recent reports showing that shift towards predominant innate immunity resulted in epigenetic changes by a high-fat diet such that their inflammatory signature persisted even after weight loss and normalization of metabolism^66^.

We demonstrated that shifts in the position of each immune cell from subdominant to dominant and vice versa was not merely quantitative, rather, new position induced new function that created a new collective function. Therefore, analysis of each immune cell type without detecting the dominant-subdominant immune cells relationship that shapes the immune pattern, could result in contradictory reports on dynamic function of each immune cell type during the course of diseases. Such dynamic function has been documented as Th17 heterogeneity^67^, CD8^+^ T cell plasticity^68^, dynamic plasticity of macrophages^69^, site-specific DC plasticity^70^, as well as immune evasion mechanisms^71,72^. Very recent review of literature on the immune system also demonstrated how immune cell metabolites affect immune cell communication and function as a system^73^.

These data provide multiple novel insights on how the impact of immune-inflammation and oncogenic pathways may vary based on the biological context and immunological pattern in which they are activated. They support future integrated approaches to evaluation of the hepatic immune patterns and their mechanism of action as the collective function that may not be evident by conventional reductionist approaches. The pattern discovery approach suggests that progressive NAFLD or NASH and HCC cannot be described by tumor immune tolerance or immune suppression, because these mechanisms focus on each cell component, which do not reflect the collective immune function. In fact, focusing on each immune cell type, pro- or anti-inflammatory cells, would mask and miss important functional mechanisms and cellular dynamics, which can only be detected by the discovery of the hepatic immunological patterns creating collective functions. Based on the present data, we suggest that taking a systems immunology approach could change the direction of immunology research, and immunotherapeutic development. We offer pattern discovery approach for the understanding of networks of immune cell interactions, rather than getting lost in the details, as well as immune modulation strategies, rather than inducing or boosting a specific type of the immune response, for cancer therapies. To this end, the hepatic structural cells, fibroblasts and endothelial cells, in addition to hepatocytes appear to dominate the outgoing signals affecting the innate immune cells, macrophages and monocytes, as well as the adaptive immune cells, T and B cells. Also, innate immune cells appear to be dominant in signaling networks compared with T and B cells when truncated mean of 50% was used. Nevertheless, a truncated mean of 2.5% revealed an active role of T and B cells in signaling network as well. Overall, innate immune cells appear to have more contribution in signaling network than T and B cells. It is important to note that the dominant-subdominant pattern is just one factor that can influence the collective immune function, feedback loops among immune cells as well as between immune cells and the hepatic structural cells or liver-resident microbiome could also participate in shaping the collective immune function in the liver. Therefore, future studies should include multi-omics studies to achieve more accurate understanding of the hepatic immunological pattern by considering the role of tissue microbiome dysbiosis in remodeling the hepatic structural cells and immunological pattern^74^. The hepatic immune patterns that we discovered in the present study are limited to the methodology we used including SingleR containing reference database for annotating major cell types, and scSorter. Further analyses may be needed by means of more advanced programs to acheive a comprehensive understanding of the hepatic immunological patterns.

## Materials and methods

### Mice and specimens

Six snap-freeze liver samples collected from DIAMOND mice (Diet-induced animal model of non-alcoholic fatty liver disease; 129A1/SvIm and C57BL/6 J cross mice) were subjected to single nuclei RNA sequencing (snRNAseq). All mice were males and stratified into three groups, each including two samples. The control group was put on a standard CD for 40 weeks (Ctrl), two experiment groups were put on a WD either for 40 weeks when they were yet to develop HCC; thus, being classified as the pre-tumor (Pre-T) group, and those who were on a WD for 48 weeks by the time they have developed tumors (Post-T). WD consists of a high-fat food diet, coupled with sugar water (23.1 g/L of Fructose and 18.9 g/L of Glucose adjusted to 1L in distilled water) to promote the diet-induced phenotype in a longitudinal fashion over time. We utilized these specific time frames on a WD based on a previous study that found male DIAMOND mice consistently developed liver tumors after 48 weeks^26^. Lastly, the snap-freeze liver samples underwent snRNAseq through the 10 × Genomics Chromium system. Specifically, the Singulomics Corporation conducted library construction with Next GEM v3.1, then libraries were sequenced with approximately 200 million paired-end and 150 base-pair long reads per sample on an Illumina NovaSeq sequencer. These studies have been reviewed and approved by the Institutional Animal Care and Use Committee (IACUC) at Virginia Commonwealth University on animal protocol number AD10001306. All methods were performed in accordance with the relevant guidelines and regulations.

### ARRIVE guidelines

These studies were also conducted in accordance with ARRIVE Guidelines, in which more detailed information can be found in the ARRIVE Essential 10 (Table 1). Previous reports demonstrated validity of deep sequencing results in small cohorts of even 2 mice per group by presenting aggregate data^75–77^.

**Table 1:** Experimental design.

ARRIVE essential 10		
Study design	1	All mice were male and stratified into three groups housed in different cages: those on a standard chow diet (CD) for 40 weeks served as a control, while the rest of the mice were fed a high-fat diet in a longitudinal fashion. Mice in the Pre-T group were on this diet for 40 weeks, while those in the Post-T group were on the diet for 48 weeks and developed HCC, as shown in the development of this DIAMOND mice model^26,27^
Sample size	2	The total number of DIAMOND mice used was six, with two mice stratified in each group (Ctrl n = 2, Pre-T n = 2, and Post-T n = 2) in order to capture cellular changes in the liver during HCC progression. Sample size was decided based on cost of sequencing services and our ability to perform a base level of statistical analyses
Inclusion and exclusion criteria	3	No animals were excluded from this study, nor was an exclusion criterion established
Randomization	4	No randomization occurred, as all mice were housed in cages on the same rack, and their cage location remained the same throughout the study
Blinding	5	No blinding was performed, as knowledge of which cage the mice were in was required to provide them with the correct experimental diet and water (CD and regular water compared to high-fat diet and sugar water)
Outcome measures	6	Any behavioral changes in mice were monitored throughout the study. The outcome measure assessed was examination of the livers from our experimental groups of mice via snRNA-seq
Statistical methods	7	DESeq2 processed data was filtered on a *p*-value < 0.01, followed by subsequent filtering in IPA of only molecules with a z-score > 2. Quantification of specific molecules of interest was performed in each replicate (n = 2 per group) in order to generate an average and standard error mean (SEM) for plotting. CellChat analyses only presented predicted signaling pathways deemed significant by the program (*p*-value < 0.05)
Experimental animals	8	DIAMOND mice (129A1/SvIm and C57BL/6J cross mice) were all males in this study and started on their respective diets at two months of age for 40–48 weeks
Experimental procedures	9	No experimental procedures were conducted during the time on high-fat diet. At the end of the time course mice were humanely euthanized to snap-freeze resected livers and subjected to snRNA-seq
Results	10	Analyses of snRNA-seq data assessed both replicates (n = 2 per group) pooled together for visualization of data in Seurat and cell type annotation. Replicates were separated in R for statistical measures during analysis with DESeq2 and quantification of cells expressing specific molecules of interest

Detailed methods and procedures utilized for obtaining and analyzing in vivo experimentation in mice.

### Hematoxylin and eosin staining

Formalin-fixed paraffin-embedded liver (FFPE) liver tissues were subjected to hematoxylin and eosin (H&E) stain using Tissue Tek Prisma Autostainer. We used VectraPolaris 1.0 acquisition software, image size of 16.76 mm × 30.18 mm with the resolution of 25 um/pixel (40 ×).

### Data pre-processing, quality control, and visualization

After sequencing, reads were aligned to the mm10 version of the *Mus Musculus* reference genome provided by 10X Genomics with CellRanger v6.1.1 to generate our single nuclei sequencing results. All the sample data was processed in Seurat R package by first giving group identifying labels to each sample, which were all merged to undergo quality control and normalization by filtering on nFeature_RNA > 200 & nFeature_RNA < 5000 and the percentage of mitochondrial gene expression < 5% (percent.mt). Too few or too many features may be indicative of dead cells or multiple cells in a single run; while increasing amounts of mitochondrial associated genes corresponds with dying cells. The nCount_RNA metric in Seurat was not included with other quality control metrics, as we did not use this for filtering steps. Initial visualization through dimensional reduction was performed on all cells within sample groups by using marker genes from SingleR cell type annotations in principal component analysis (PCA), followed by uniform manifold approximation and projection (UMAP) (Fig. S1C; arXiv:1802.03426). Graphical visualization of cell clusters was performed with both PCA (Fig. 1B) and UMAP (Fig. 1C,D) in Seurat, followed by functional interrogation of cell types through additional R packages. Also, the Viridis package was utilized for improving heatmap coloration when using Seurat’s DoHeatmap function to assess any genes of interest^78^; along with the pheatmap package for the reference annotation probability score heatmap (Fig. S1C).

### Reference database cell type annotations and cellular subset quantification

Cell typing was performed using a 2-pass method. First, we used SingleR^79^, which enables access to reference databases of annotated cell types from a multitude of different experiments, in order to annotate cell types globally by using both ImmGen and MouseRNAseqData databases. This was performed to ensure accuracy of cell type annotations, which was confirmed through matched probability scoring of annotated cells by both databases (Fig. S1C). Only matched cell types with the same nomenclature were compared, as combining the use of both databases enabled us to identify cell types unique to one database or the other, such as Hepatocytes or NKT cells (http://bioconductor.org/books/release/SingleRBook/using-multiple-references.html). The ImmGen database contains 830 microarray samples focused on the classification of 20 main cell types and various subtypes of hematopoietic and immune cells, while the MouseRNAseq database contains 358 RNA-seq samples focused on annotating 28 specific cell types. All cell types annotated with both databases had the top 20 markers of each cell type recorded, so we could pass all gene markers excluding duplicates to PCA to optimize downstream visualization through dimensional reduction methods. All marker genes can be found in an Excel file listed in the Table 2 (SingleR Annotated Cell Markers xlsx file). Second, we used scSorter^80^, which performs a “semi-supervised” machine learning approach, to further interrogate subsets of our singleR annotated cell types, such as T cells, Dendritic cells (DCs), B cells, Monocytes, and Macrophages. All cells were sorted based on known marker genes, along with non-marker genes using the scSorter R package, which employed a machine learning approach to differentiate the subsets of interest for downstream analyses. DCs were sorted into DC1, DC2, and pDC; Macrophages were sorted into M1, M2, M1-like, and Kupffer cells; Monocytes were sorted into monocytic-MDSCs (mMDSC) or remained classified as monocytes; and T cells were sorted into CD4 and CD8 subsets. The CD4 T cell subset was further interrogated by classifying helper T cell subsets (Th1, Th2, Th17, and Treg) with scSorter to assess their functional pattern in each sample group. We initially sorted CD4 and CD8 T cells with marker genes from “GeneList18”, which recapitulated patterns seen in previous works showing different ratios in the spleen compared with the liver (Mirshahi et al., 2022), so we reported it in Fig. 1H. However, we sorted CD4 and CD8 T cells differently with “GeneList14” in order to interrogate helper T cell subsets, as we needed to acquire a higher number of cells with the algorithm to sufficiently capture the pattern of helper T cells on WD. These specific sorting results were only included in the UMAP visualization on Fig. 1D, demonstrating their pattern in Fig. 1G, and in Fig. 5 to investigate specific cellular sources of signaling. All sets of marker genes to sort cell type subsets can be found in an Excel file within Table 2 (IC Patterns scSorter genes and PMIDs xlsx file). All cells classified by SingleR annotations and scSorter were first quantified in R to understand the exact number of each cell type per sample group, and further recorded in Microsoft Excel for normalization and graphing. Any “Unknown” cell populations from the use of scSorter occur because they do not fit into any of the subsets of interest based on gene expression, so these were removed from analyses if there were few cells (n < 10 in any of the three groups). If there were sufficient cells, they were interrogated for any specific differentially expressed genes that may implicate an identity. M1-like cells were classified based on their similar expression profile to M1 cells, especially their high expression of Itgad, a marker of pro-inflammatory macrophages retained in inflamed sites^33–35^ (Fig. S2). When sorting CD4 + Helper T cell subsets, we sorted differently with “GeneList18” markers to optimize the number of CD4 + T cells to sort out the helper T cells. This was performed to find the proportion of the pattern that helper T cells constitute on WD and to identify potential sources of signaling with the CellChat R package downstream. Notably, this group did have significantly more than 10 cells classified as unknown in one group (Ctrl: n = 98, Pre-T: n = 21, and Post-T: n = 9). However, because the Post-T had less than 10 cells and differential expression analysis in Seurat did not reveal any distinguishing genes compared with other helper T cell subsets, these cells were removed to ensure only accurate annotations were included downstream. All known classified cellular subsets were normalized to 100% based on the total cells within that cell type category excluding any “Unknown”.

**Table 2:** Marker genes table. SingleR Annotated Cell Markers contains the top 20 marker genes for each cell type based on the SingleR reference databases, in which all unique and duplicate genes were compiled to pass these genes to principal component analysis.

Marker genes table	
SingleR cell type PCA genes	SingleR annotated cell markers xlsx file
scSorter cell type subsets	IC patterns scSorter genes and PMIDs xlsx file

IC Patterns scSorter genes and PMIDs contains marker genes for sorting cell type subsets out of the SingleR annotated population, in which the “GeneList# file for R” column shows the set of markers used for identifying subsets in each annotated cell type; along with PMIDs supporting the use of specific marker genes. All files can be found in the repositories listed in the data and code availability section.

### Differential gene expression

Differential gene expression analysis was performed with DESeq2^81^ to identify differentially expressed genes across sample groups to compare expression signatures by uploading data containing the calculated log fold change and statistics of group comparisons to Ingenuity Pathway Analysis (IPA). Specifically, three groups were stratified to assess disease progression: first comparing Pre-T cells to those in the Ctrl (Ctrl vs. Pre-T), Post-T cells against those in the Ctrl (Ctrl vs. Post-T), and finally the Post-T group compared against the Pre-T (Pre-T vs. Post-T). A pooled or bulk immune cell analysis was performed by consolidating all immune cells into a single Seurat object within each experimental group to assess any differences we see in a collective immune signature, compared to the individual SingleR annotated cell populations we performed DESeq2 on alone. Certain cell types were combined to ensure sufficient counts were present for statistical comparison, such as NK and NKT cells (NK/NKT), along with the remaining innate immune cells such as Microglia (microglia-like), Basophils, Eosinophils, Granulocytes, Neutrophils, ILC, and Tγδ cells (other innate cells). Since we detected microglia-associated gene expression signatures in the liver, we used the term microglia-like cells for microglia. The pooled immune cell group had counts of greater than or equal to 10 when running DESeq2, whereas investigation of individual cell populations used a filter of greater than or equal to 3 to accommodate for sample size differences. Figure 3 took another pooled approach by merging all annotated and sorted cell type subsets to analyze the hepatic microenvironment in each group (includes: B1, B2, pro-B cells, CD8 T, Th1, Th17, Th2, Treg, NK, NKT, Tγδ, DC1, DC2, pDC, m-MDSC, Monocytes, Kupffer, M1, M2, M1-like, Microglia (microglia-like), Basophils, Eosinophils, Granulocytes, Neutrophils, ILC, Fibroblasts, and Hepatocytes).

### Identification of intercellular signaling networks

The recently developed R package called CellChat^82^ employs an online manually curated database based on KEGG and various published articles to generate a comparative framework for detecting intercellular communication across biological conditions. It characterizes and compares inferred signaling networks using a novel approach that analyzes through looking at social networks, pattern recognition, and manifold learning. This approach enables evaluation of all predicted signaling networks between specific cell types, while also identifying sources and targets of signaling interactions based on gene expression of the ligand-receptor pairs between cell populations in a sample group. We performed this analysis with just our SingleR annotated immune cells, along with the annotated non-immune cells of interest such as hepatocytes and structural cells (Fig. 4A). This was followed by a second perspective of analysis, in which only SingleR annotated immune cells were included to assess any immune-immune cell signaling interactions during disease progression (Supplemental Fig. S5). Cells were also filtered so only groups with more than 10 cells were assessed to ensure accurate representation of intercellular communication networks between cell types. Further, we performed multiple layers of this analysis with CellChat by adjusting the parameters in the “computeCommunProb” function to a “truncatedMean”, in which we were able to examine the ligand-receptor pairs detected in a specific percentage of cells within each SingleR annotated cell type, such as 50%, 25% (Default “trimean” approach), 5%, and 2.5% to detect even lowly expressed interactions in immune cells.

### Enrichment analysis

Results from DESeq2 were uploaded to IPA (QIAGEN Inc., https://www.qiagenbioinformatics.com/products/ingenuity-pathway-analysis), which enabled an in-depth interrogation of functional differences based on counts between cell populations of interest compared across sample groups. All cell type populations analyzed through DESeq2 were filtered on a *p*-value less than 0.01, and subsequently a z-score greater than 2 in the IPA analysis results. Enrichment was primarily assessed for terms such as cancer and disease related functions, signaling pathways, immune response, cytokines, microRNA (mir-RNA), and metabolism.

### Quantification and statistical analysis

All cells (SingleR annotated and scSorter identified subsets) were quantified in R and recorded in Microsoft Excel, in order to normalize the data for immune and non-immune cells, respectively. Each group was treated as a single sample, as n = 2 for each experimental group. However, all DESeq2 results were filtered in IPA to only assess genes with a *p*-value < 0.01, followed by subsequent filtering of IPA analysis results for detected functions and molecules with z-scores > 2. All cell types expressing specific genes of interest identified in our CellChat analyses (TNF-α, ADAM17, TNFR1, TNFR2, and double positive cells) were identified in Seurat and quantified to differentiate how TNF signaling is occurring in a more mechanistic fashion within these cell types (Fig. 4C). Further, extracting each set of predicted signaling interactions from our CellChat analyses enabled us to focus on signals stemming from structural cells in Fig. 5, and the probability scores of those interactions with other cells in the liver were graphed for each signaling interaction to see which cell type was highly predicted to be receiving the signal.

### Supplementary Information

Supplementary Figures.

## Data Availability

All code has been deposited on GitHub (https://github.com/koelschnj/Hepatic-Immune-Cell-Patterns-Code) and is publicly available on the following link: https://www.ncbi.nlm.nih.gov/geo/query/acc.cgi?acc=GSE225381. The datasets generated and/or analyzed during the current study are available in the GEO repository, GSE225381.

## Materials availability

DIAMOND mice used in this study will be provided, upon request to the lead contact, and may require fulfillment of an MTA. This study did not generate new unique reagents.
