# Supplementary material for: The crosstalking immune cells network creates a collective function beyond the function of each cellular constituent during the progression of hepatocellular carcinoma
Source: Sci Rep. 2023 Aug 3;13:12630. doi: 10.1038/s41598-023-39020-w (PMC10400568; doi:10.1038/s41598-023-39020-w)
Supplement: Supplementary file 1 — Supplementary Figures. [file 41598_2023_39020_MOESM1_ESM.pdf]

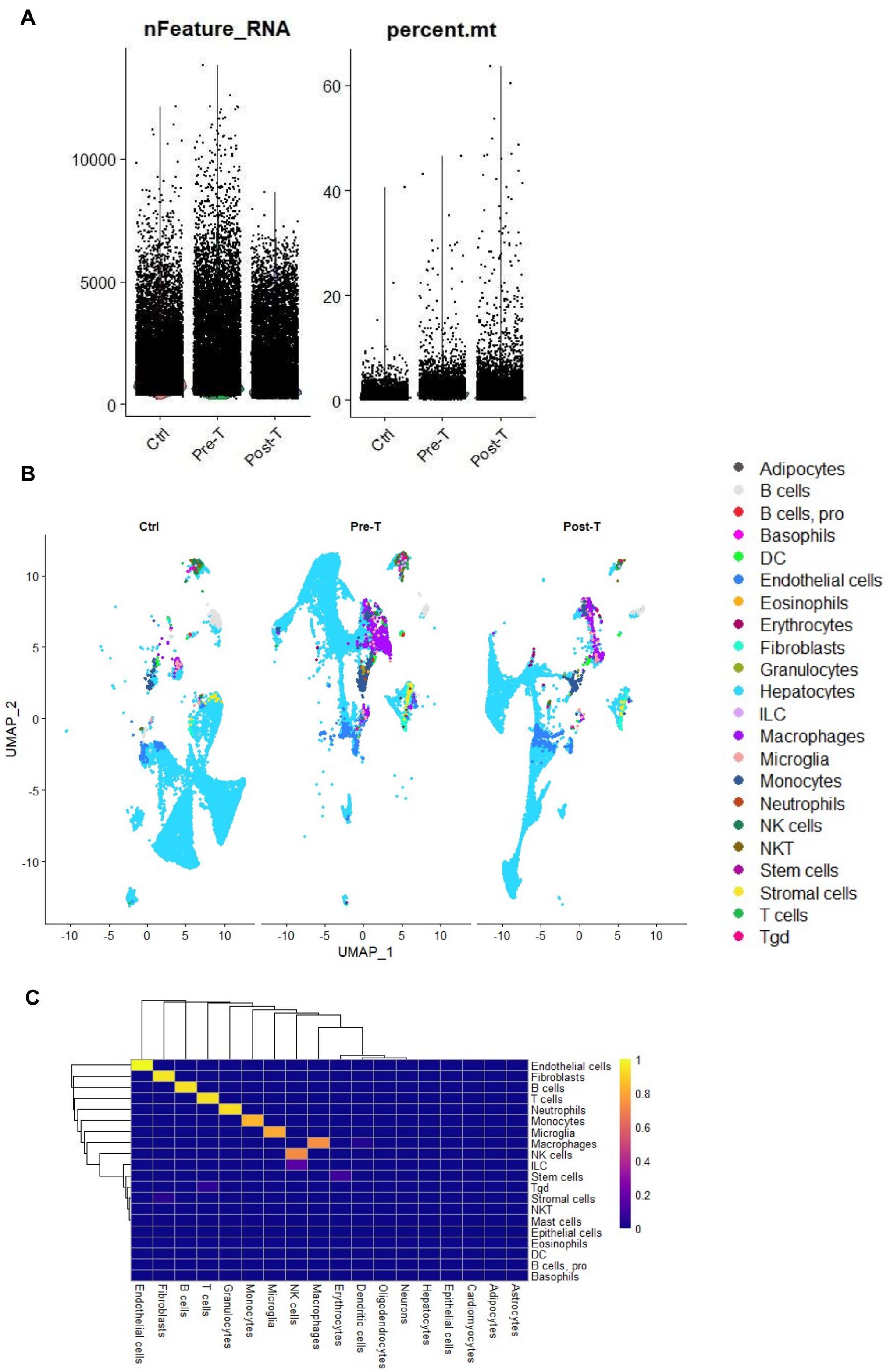

Figure S1. Normalization and cellular annotation of the liver: A) Quality control metrics employed to use only high-quality cells (cells were filtered on nFeature_RNA > 200 & nFeature_RNA < 5000 & percent.mt < 5. B) UMAP portraying cell type clusters in experimental sample groups separately after SingleR annotation of cell types. C) Heatmap portraying all matched cell type annotation probability scores based on the two murine reference databases (Immgen and mouse RNAseqdata) of annotated cells (probability score close to 1 indicates exact matches, whereas cell type annotations that are not shared between both databases were not compared). All cell type nomenclature on each axis stems from each individual database, so we could not alter the naming scheme, such as microglia, which will be termed “microglia-like” throughout the rest of the manuscript.

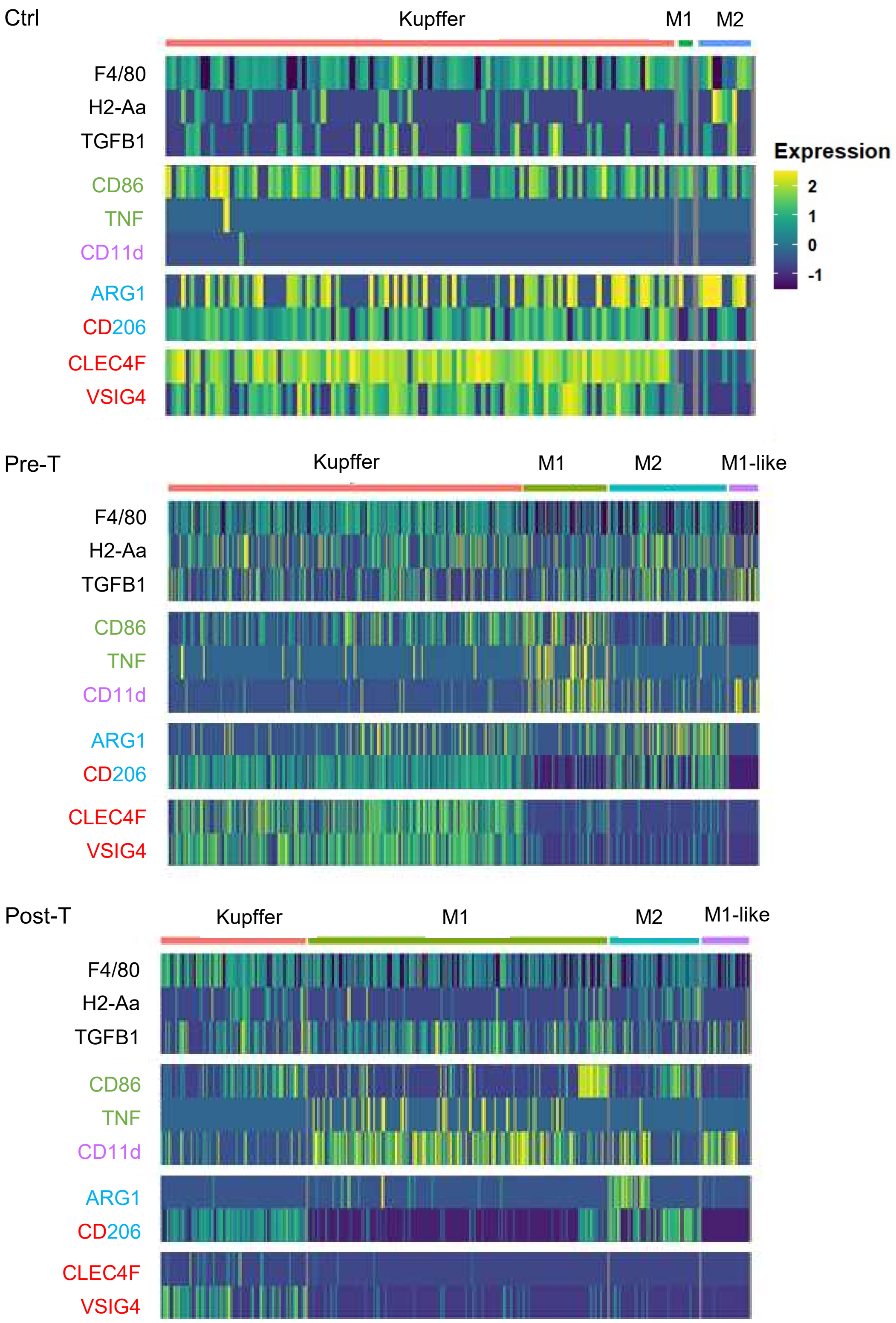

Figure S2. Macrophage functional profiles: Heatmaps showing the gene expression profile of major macrophage subsets (Kupffer cells, M1, M2, and M1-like) across groups (size of heatmap bands corresponds to the proportion of cells found with macrophage population by scSorter algorithm, where font color of genes on left of heatmaps corresponds to color above heatmaps and markers used to identify the subsets). All heatmaps in this figure were generated through the DoHeatmap function in Seurat version 4.3.0 (https://cran.r-project.org/web/packages/Seurat/index.html) and the viridis package version 0.6.2 for coloration (https://cran.r-project.org/web/packages/viridis/index.html).

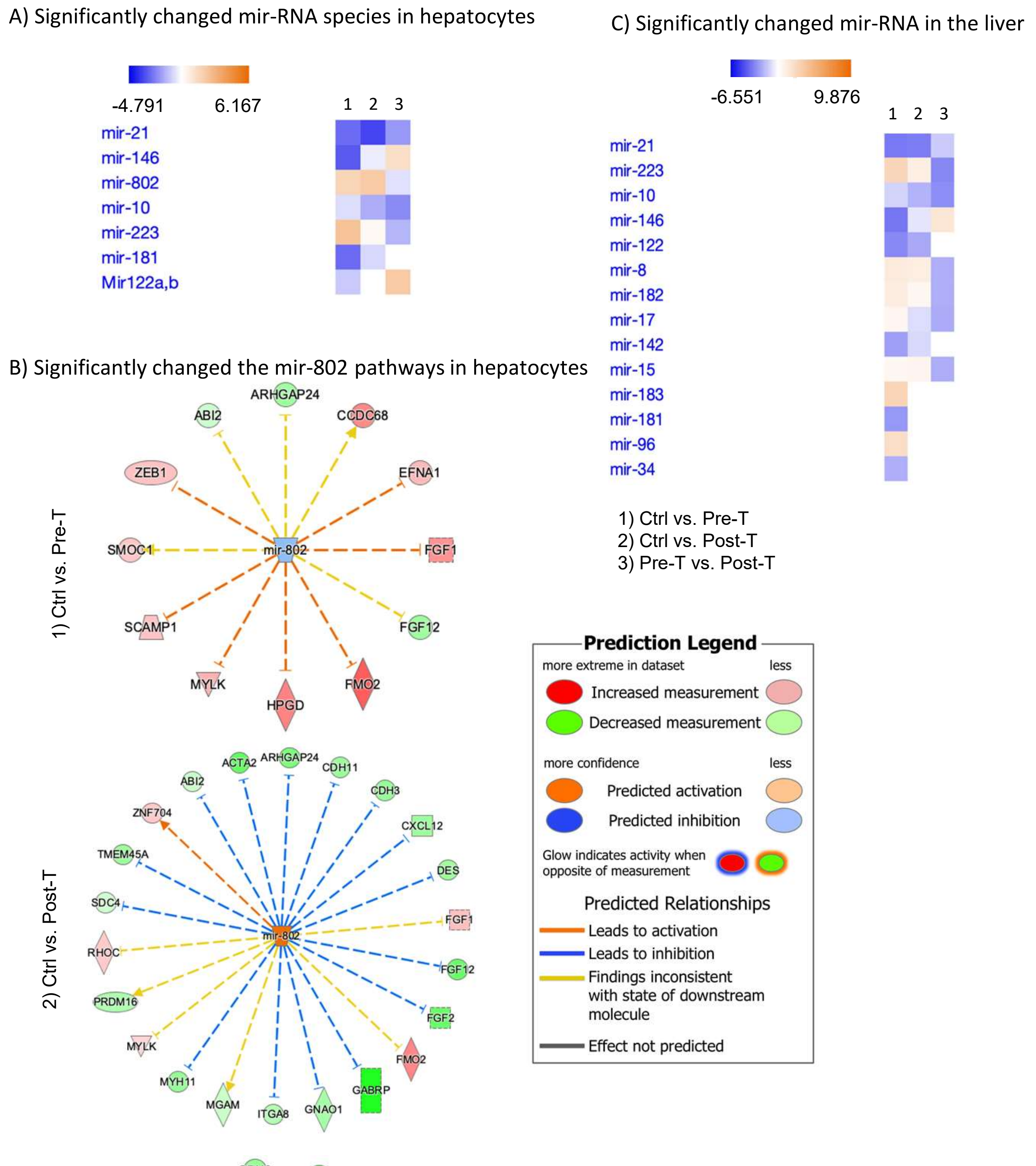

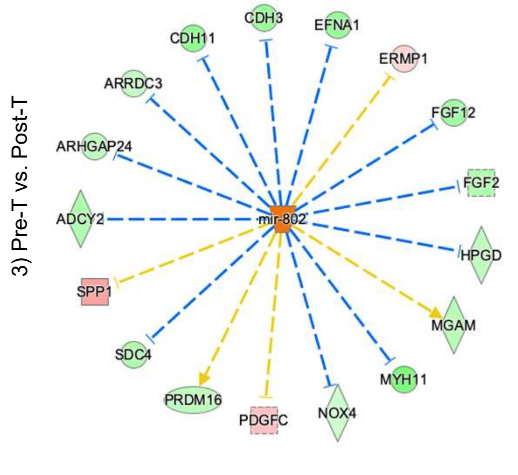

Figure S3. Detection of significantly changed micro-RNA expression in hepatic cells: A) Significant upregulated or downregulated mir-RNA species detected in hepatocytes comparing three groups. B) Significantly changed pathways linked to mir-802 in hepatocytes. C) Significantly upregulated or downregulated mir-RNA detected in pooled SingleR annotated and scSorter identified subsets across groups.

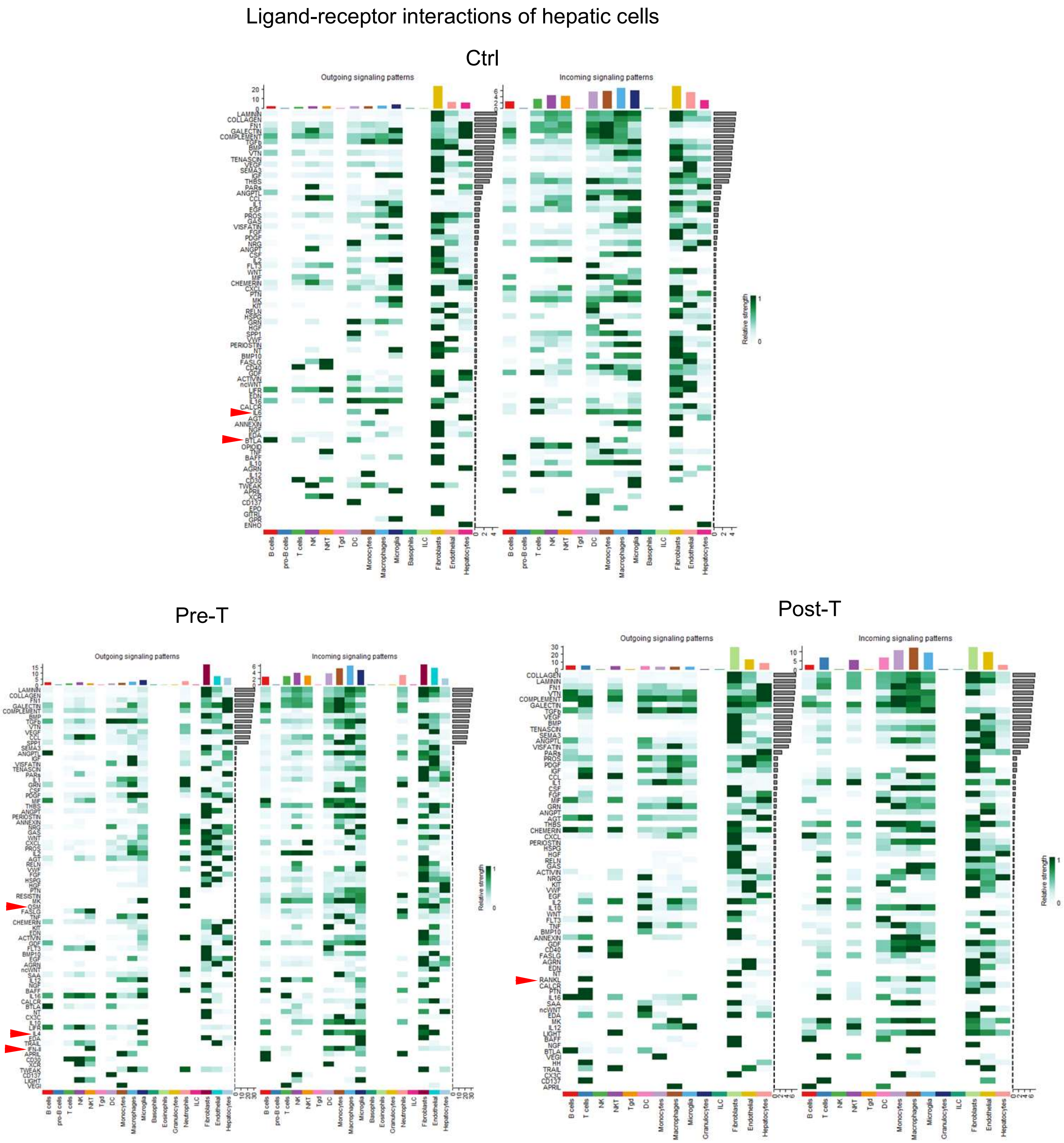

Figure S4: CellChat Signaling analysis of hepatic cells. CellChat analyzed cell type specific ligand-receptor signaling interactions in our SingleR annotated immune cells, structural cells, and hepatocytes. The analysis was conducted with truncated mean = 2.5% in order to detect even lowly expressed critical immunologically relevant signaling pathways. These heatmaps show all significantly detected signaling pathways in the Ctrl (upper panel), Pre-T (lower left panel), and Post-T (lower right panel). All heatmaps were generated through the use of CellChat version 1.5.0 (https://github.com/sqjin/CellChat), and the dependent software ComplexHeatmap version 2.15.1 (https://github.com/jokergoo/ComplexHeatmap).

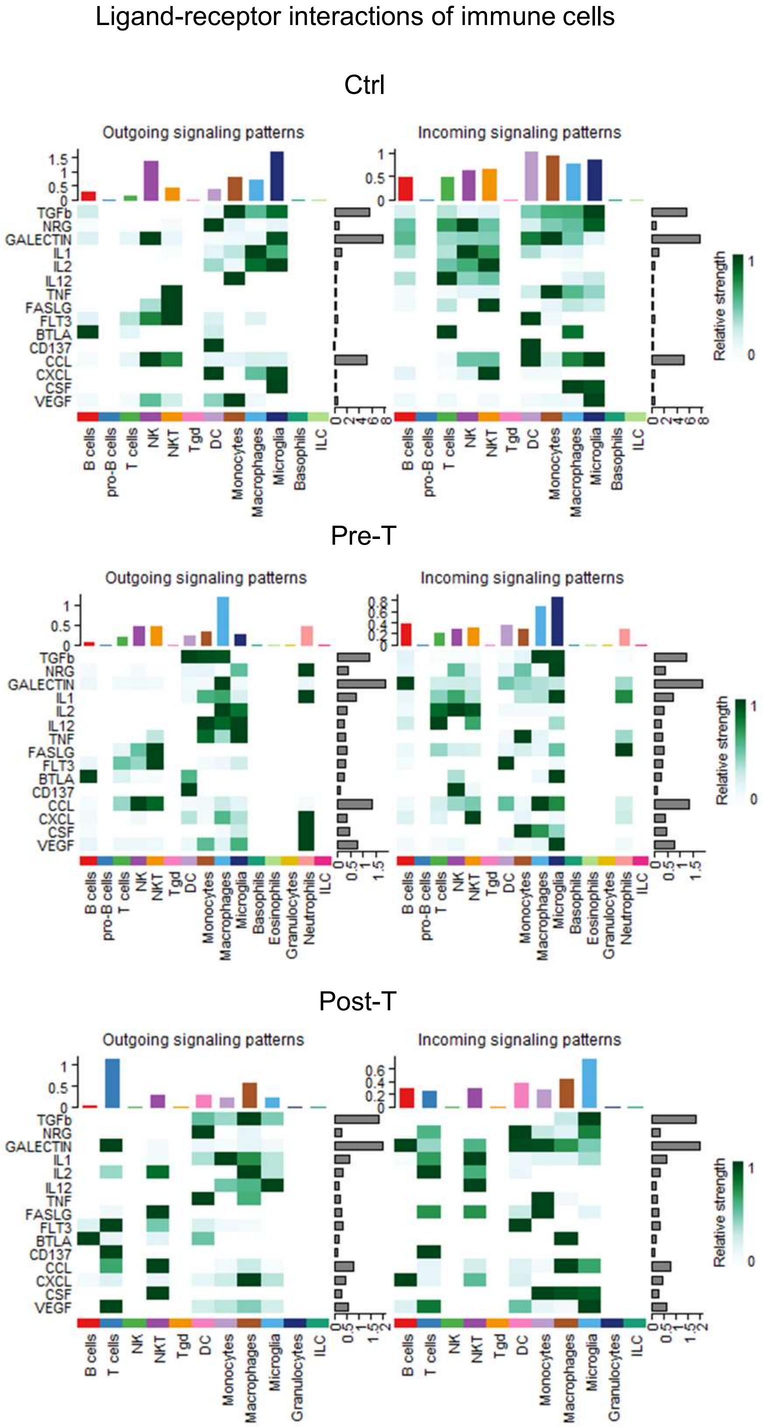

Figure S5: CellChat Signaling Analysis of immune cells in the liver. Another perspective of signaling was assessed by only using SingleR annotated immune cells, rather than including structural cells and hepatocytes. Once again, we adjusted CellChat parameters to use a truncated mean = 2.5% to detect additional pathways of interest. Here we show key shared immune cell pathways (TGF-β, NRG, Galectin, IL-1, IL-2, IL-12, TNF-α, FASLG, FLT3, BTLA, CD137, CCL, CXCL, CSF, and VEGF) across the Ctrl (upper panel), Pre-T (middle panel), and Post-T (lower panel) groups. All heatmaps were generated through the use of CellChat version 1.5.0 (https://github.com/sqjin/CellChat), and the dependent software ComplexHeatmap version 2.15.1 (https://github.com/jokergoo/ComplexHeatmap).
